# Predictor's of Mortality in Patients with Aneurysmal Subarachnoid Haemorrhage and Reebleding

**DOI:** 10.1155/2015/545407

**Published:** 2015-02-05

**Authors:** Dannys Rivero Rodríguez, Claudio Scherle Matamoros, Leda Fernández Cúe, Jose Luis Miranda Hernández, Yanelis Pernas Sánchez, Jesús Pérez Nellar

**Affiliations:** ^1^Stroke Unit Department, “Cmdt. Manuel Fajardo” Hospital, 10600 Havana, Cuba; ^2^Stroke Unit Department, “Ameijeiras Brother's” Hospital, 10300 Havana, Cuba; ^3^Internal Medicine Department, “Calixto García” Hospital, 10400 Havana, Cuba

## Abstract

*Methods.* “Ameijeiras Brother's” and “Cmdt. Manuel Fajardo” Hospitals enrolled 64 patients (multicentre retrospective cohort) with aneurysmal subarachnoid haemorrhage and rebleeding. The patients were admitted to the Stroke Unit (SU) between January 1, 2006, and December 1, 2013. Demographic, clinical, and radiological variables were examined in logistic regression to evaluate independent factors for increasing the risk of death. *Results.* Patients with systolic blood pressure >160 mmHg (*P* = 0.02), serum glucose >7 mmol/L (*P* = 0.02), aneurysm location in artery communicant anterior (*P* = 0.03), and black/mixed race (*P* = 0.008) were significant related to death in univariate analysis. Risk factors (HTA, smoke, alcohol consumption, and DM), complication, multiplex rebleeding and stage of WFNS, and Fisher's scale were not related to mortality. Patients with three or more complications had a higher mortality rate (*P* = 0.002). The results of the multivariate logistic regression analysis indicated that race (black/mixed, *P* = 0.00, OR 4.62, and 95% IC 1.40–16.26), systolic blood pressure (>160 mmHg, *P* = 0.05, OR 2.54, and 95% IC 1.01–3.13), and serum glucose (>7.0 mmol/L, *P* = 0.05, OR 1.82, and 95% IC 1.27–2.67) were independent risk factors for death. *Conclusions.* The black/mixed race, SBP, and serum glucose were independent predictors of mortality. Three or more complications were associated with increasing the probability to death. Further investigation is necessary to validate these findings.

## 1. Background

Aneurysmal Subarachnoid Hemorrhage (aSAH) is a devastating condition with high mortality and morbidity rates for those who survive the initial haemorrhage. Population-based study informed mortality rates range from 8% to 67% with a significant morbidity among survivors [1]. Many survivors have neurologic deficit that notably limits their physical and mental status. In reality, only a few patients can return to their normal daily living activities as carried out before the haemorrhage [2]. Nonetheless two-thirds of the patients with aSAH regained functional independence and increased the survival rates in 17% in the last years [3].

Rebleeding has been recognized as a leading preventable cause of death and disability after aSAH. Mortality is reported to be as high as 80% in patients with rebleeding [4, 5]. Whereas rebleeding contribution to morbidity and mortality is well established, the mechanism by which rebleeding drives poor outcomes is not yet known [6].

Some factors has been related with mortality in aSHA, female sex, severity of clinical presentation, rebleeding, older age, preexisting severe medical illness, global cerebral edema on computed tomography (CT) scan, intraventricular and intracerebral haemorrhage, symptomatic vasospasm, delayed cerebral infarction (especially if is multiple), hyperglycemia, fever, anaemia, and other systemic complications such as pneumonia and sepsis [7]. Nevertheless, when rebleeding is present the conditions that predict mortality are not well established.

The aim of this study is to evaluate the admission factors predicting hospital mortality in patients with rebleeding after an aSAH.

## 2. Methods

### 2.1. Patients Population

We retrospectively reviewed patient-related data from a prospectively collected database of patients with Rebleeding after aSAH admitted to “Ameijeiras Brothers” and “Cmdt. Manuel Fajardo” Hospital between January 2006 and December 2013. An amount of 64 patients was included in analysis. They met the following criteria: (1) ≥18 years of age; (2) SAH that was established on the basis of admission computed tomographic (CT) scans or by xanthochromia of the cerebrospinal fluid; (3) the aneurysm that was held responsible for the hemorrhage which was demonstrated by CT-angiography (CTA) and/or digital subtraction angiography (DSA); (4) grade 4 or menus in scale World Federation Neurologic Surgeon (WFNS) [8]; (5) eight or more points in Glasgow Coma Score [9]; (6) rebleeding that was defined by repeated CT scans presenting an increase of subarachnoid bleeding, intracerebral, or intraventricular hematoma and by changes noted in documented clinical signs or unexpected increase headache or sudden deterioration of consciousness or sudden apnea.

The exclusion criteria was: aneurysm not held, rebleeding suspected in prehospital care, death before the diagnosis protocol finished and mycotic aneurysm.

### 2.2. Data Collection

The both institutional ethics committees approved the protocol before commencement. All participants provided informed consent, including next of kin for patients who were severely ill, unconscious, or obtunded and including proxy respondents.

Information on demographic characteristic (age at diagnosis, sex, and race) and risk factors was collected. Hypertension and diabetes were defined as a history of treated hypertension or/and antidiabetes drugs. Smoking status was classified into current smokers and nonsmokers (including exsmokers and those who had never smoked). Alcohol consumption was considered positive when patients or family mentioned that patient takes 350 mL of Rum or more than six bottles of beers at the week by one month consecutively in the last three months.

### 2.3. Clinical Variables

Clinical conditions were registered according the WFNS scale at hospital admission. Functional outcomes at hospitality discharge was assessed with the modified Rankin Scale (mRS), a global disability scale with scores that range from 0 (no symptoms) to 6 (death) [10]. The admission blood pressure (BP) values were obtained during the first 8 hours after the patient arrived in the stroke unit by a trained nurse using a calibrated sphygmomanometer. For all patients, at least eight BP readings were made during this time. Out of these, the highest value was chosen as the admission BP. The mean BP was calculated by the fallowing formula (2  diastolic  BP + systolic  BP)/3. Patients were classified >160 mmHg, ≤160 mmHg and value of blood pressure was also registered. Similar description was used to serum glucose value (>7 mmol/L and quantitative value) and measurement was taken at the stroke unit admission.

Some complications were recorded moreover: pneumonia, sepsis, Hidroelectrolite disorder, hydrocephalus, and symptomatic vasospasm. Rebleeding day was considered to take into account day zero refers to the calendar day of the onset SAH, defined as the clinical bleeding event that immediately preceded emergency room admission.

### 2.4. Radiological Variable

The amount of subarachnoid blood on the admission CT scan was graded according to Fisher scale [11]. Multiplex rebleeding was defined as a CT scans presenting more increase of subarachnoid bleeding than the previous study, after the first event of rebleeding. Aneurysm location was registered also. All neurovascular studies was analyze by the same group of vascular neurology and neuroradiology of the institution.

### 2.5. Clinical Management

All patients were treated according to standardized protocol, which closely follows international guidelines [7, 12]. Aneurysm repair was always done as feasible as possible, preferably in first 48 hours at the admission stroke unit. The choice of treatment modality (clipping, coiling, or none) was made in consensus between neurologists and neurosurgeon. Usually an endovascular technique was performed in posterior circulation aneurysm and aneurysm of anterior circulation was clipping, but all cases were discussed in staff meeting and treatment was specific for each patients. All patients received 0.9% normal saline at a dose of 1 mL/kg/hour, and supplemental 5% albumin solution was administered to maintain central venous pressure at 5 mmHg if needed. Standard medications are nimodipine, acetaminophen analgesia, supported with additional analgesics when necessary, antiepileptic drugs, and laxatives. An external ventricular drain (EVD) was placed in all patients with symptomatic hydrocephalus or intraventricular haemorrhage with depressed level of consciousness. Transcranial Doppler (TCD) was performed daily.

### 2.6. Statistical Analysis

Baseline characteristics, in-hospital complications, and clinical and radiological variables were compared between the group of survivors and death (Rankin Scale 6), using 1- and 2-tailed Student *t*-tests and the Mann-Whitney *U* test for continuous variables and Fisher exact test or Chi square test for categorical variables. Candidate demographic, clinical, and radiological variables in the univariate analysis were used to create a multivariable model for independent predictors of mortality using generalized linear regression. Logistic regression was used to identify independent predictors of death. Admission predictors were then added individually to these models to calculate adjusted odds ratios for the strength of association of mortality. Tests for interactions were performed for all of the significant variables in the multivariable models. A *P* value of 0.05 or less was considered statistically significant. All statistical calculations were made with widely available commercial software (SPSS 17.0, Chicago, IL).

## 3. Results

### 3.1. General Characteristic and Univariate Analysis

In total, 351 patients were diagnosed with aSAH, an amount of 64 patients rebleeding. The hospital mortality rate in the group with rebleeding was 57.81%. The mean (SD) age of the 64 patients was 50.2 (11.8) years and 51.5% were female. Small differences were found according age and sex in the groups of patients survivors and dead, 52.55 (11.87) versus 53.27 (13.4), 15 (55.55%) versus 18 (48.64%), respectively. Black/Mixed race was significantly associated with hospital mortality in univariate analysis; 23 (62.16%) of patients dead versus 7 (25.93%) survivors (*P* = 0.008). Risk factors (HTA, smoke, alcohol consumption, and DM), complication, multiplex rebleeding, stage of WFNS, and Fisher scale were not related to mortality. Patients with systolic blood pressure >160 mmHg, serum glucose >7 mmol/L, and aneurysm location in artery communicant anterior were significantly related to dead (Table 1). It is important to mention that 27 patients were admitted at the stroke unit after 72 hours since initial symptoms, 8 patients had bad clinical conditions, 6 patients had late diagnosis of the aneurysm, and 5 patients taken complex aneurysm. Reason why several patients received late neurosurgical treatment.

### 3.2. Multivariable Analysis

The systolic blood pressure range was 144 ± 26.2 mmHg for all patients. The analysis reflected that the systolic blood pressure and serum glucose were significantly higher in dead group (SBP *P* = 0.034) (serum glucose *P* = 0.014) (Table 2).

The results of the multivariate logistic regression analysis indicated that race (Black/Mixed *P* = 0.00, OR 4.62, 95% IC 1.40–16.26), systolic blood pressure (>160 mmHg *P* = 0.05, OR 2.54, 95% IC 1.01–3.13), and serum glucose (>7.0 mmol/L *P* = 0.05, OR 1.82, 95% IC 1.27–2.67) were independent risk factors for dead (Table 3).

### 3.3. Complication

**Figure 1:**
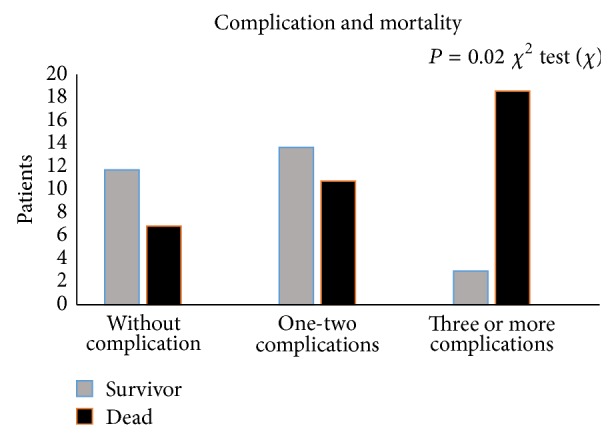
Complication and mortality.

The number of complications was significantly related to mortality. Patients with three or more complications (pneumonia, hydrocephalus, symptomatic vasospasm, Hidroelectrolite disorders, seizure, and sepsis) had a higher mortality rate (*P* = 0.002) (Figure 1).

## 4. Discussion

Aneurysm rebleeding is associated with very high mortality and poor prognosis for functional recovery in survivors. When modified Rankin scale was used the rate of persistent dependence was between 8% and 20%. Mortality rates vary widely across published epidemiological studies, ranging from 8% to 67% [1]. Mortality rates of patients with rebleeding in these series were similar to previous report (57.81%), although the sample of this research only included patients with aSAH and rebleeding. The authors did not found preceding investigations that emphasis rebleeding patient merely.

Several factors are related through mortality and poor outcome in aSAH (rebleeding, hydrocephalus, symptomatic vasospasm, older age, black people, female, intraventricular and intracerebral hemorrhage, delayed cerebral infarction (especially if multiple), aneurysm location, hyperglycemia, fever, anemia, and other systemic complications such as pneumonia and sepsis) [13]. In this investigation the univariate analysis showed the Black/Mixed race, serum glucose level, SBP, and aneurysm location in AcomA and patients with three or more complications were associated to mortality. It is not coincidence certain relationships if we consider that all patients has both diagnosis: aSAH and rebleeding. The author's reflection is probably that these factors are related through mortality in patients with aSAH and rebleeding likewise. New investigations are needed to evaluate the independent predictor's risk factors of mortality in patients with aSAH and rebleeding.

The difference in racial survival had been disclosed in previous research [14, 15]. These studies suggest higher mortality in blacks, American Indians/Alaskan Natives, and Asians/Pacific Islanders than in whites. The results of this study agree to New York SPARC (Statewide Planning and Research Cooperative System) database that suggested that white patients with SAH had better functional outcomes than nonwhite patients [16]. However some different findings are reported by other author's [17].

SBP and history hypertension have been associated with unfavorable outcome after aSAH by Rosengart et al. [13]. Similar result was found in the present study in patients with aSAH and rebleeding, but different effects were established in 2010 by Cha et al. [18]. It is remarkable to mention that a few researches are focused on to evaluate the relation between blood pressure, rebleeding, and mortality after aSAH when it is recognized that the hypertension is important risk factors for aneurysm rupture [19]. Only five investigations were included in recent meta-analysis to examine SBP as a risk factor of rebleeding [20]. Unquestionably, more studies are needed.

Hyperglycemia has been associated with morbidity and poor outcome in patients with aSAH [21, 22]. Scholars expressed that the systemic glucose levels affect glucose availability to the brain and can impact cellular metabolism and energy production after aSAH. Because of impaired glucose transport, systemic glucose levels considered to be normal may be relatively insufficient to meet the increased cerebral metabolic demand seen in patients with SAH [23, 24].

Another point considered by experts is the acute fluctuations of systemic glucose which have also been associated with oxidative stress in diabetic outpatients, with increased mortality in critically ill patients and with worse functional outcome and mortality in neurological patients. Patients with aSAH may be more vulnerable to glycaemic variability if these acute fluctuations trigger cerebral metabolic distress and lead to secondary brain injury [24, 25]. The author does not found any other study that included patients with aSAH and rebleeding.

The cluster of complications (three or more) increases the number of dead patients, especially in patients with a severe condition such as aSAH and rebleeding. Similar findings were reported by Naval et al. in validation of the “SAH score” (S = clinical status, A = age, and H = health conditions) to predict mortality [26]. Naval found that the admission GCS, age, and presence of 1 or more of the major medical comorbidities significantly affected mortality after multivariate analysis. The group of comorbidities was defined from the Charlson index, which has been validated in the setting of ischemic stroke [27] and ICH [28]. Charlson index included congestive heart failure, myocardial infarction/coronary artery disease, peripheral vascular disease, diabetes mellitus, chronic obstructive pulmonary disease, stroke, dementia, peptic ulcer disease, liver cirrhosis, renal failure, connective tissue disease, human immunodeficiency virus/acquired immunodeficiency syndrome, and cancer. In our study three or more complications were significantly related to mortality. The neurologic complications were included in the statistical analysis predominantly.

One of the major limitations of this study is that the sample size was relatively small. Second, the hospital factors related to mortality were not included in statistics analysis. Third, the complications recorded were neurological, sepsis, pneumonia, and hidroelectrolite disorders merely. Fourth, complications consequent of medical and neurosurgical procedure were not recorded. Fifth, cardiovascular complications were not included also. Finally, this study descriptively analyzed relations of some factors and mortality in patients with aSAH and rebleeding.

## 5. Conclusions

This study found that patients with aSAH and rebleeding had higher mortality rate. The ethnic difference, SBP, and serum glucose were independent predictors of mortality. Three or more complications were associated with increase the probability to die. Further investigations are necessary to validate these findings.

## Figures and Tables

**Table 1:** Univariate analysis of characteristics of rebleeding patients after aneurysmal SAH.

Variables	Survivors (%) *n* = 27	Dead (%) *n* = 37	*P* value
Sex			0.76
Female	15 (55.56)	18 (48.64)	
Age			0.85
≤45	5 (18.51)	9 (24.32)	
46–65	19 (70.37)	23 (62.16)	
>65	3 (11.12)	5 (13.52)	
Race			0.008^*^
Black/Mixed race	7 (25.93)	23 (62.16)	
Risk factors			
HTA	20 (74.07)	28 (75.67)	0.88
Smokers	12 (44.44)	18 (48.64)	0.93
Alcohol consumption	13 (35.13)	12 (32.43)	0.31
DM	1 (3.71)	3 (8.11)	0.84
Syst. BP			0.02^*^
>160 mmHg	3 (11.12)	13 (35.13)	
Mean BP			0.91
>110 mmHg	9 (33.33)	14 (37.83)	
Serum Glucose			0.02^*^
>7.0 mmol/L	3 (11.12)	13 (35.13)	
Aneurism location			
AComA	6 (22.22)	18 (48.64)	0.03^*^
MCA	5 (18.51)	2 (5.41)	0.20
PCoA	6 (22.22)	9 (24.32)	0.91
Carotid A	2 (7.42)	3 (8.11)	0.71
Others	8 (29.63)	5 (13.52)	0.77
Complications			
Pneumonia	4 (14.81)	12 (32.43)	0.24
Sepsis	5 (18.51)	5 (13.52)	0.84
Hidroelectrolite D.	3 (11.12)	8 (21.62)	0.44
Hydrocephalus	8 (29.63)	7 (18.91)	0.48
Seizure	0 (0)	3 (8.11)	0.35
Sympt. vasospasm	4 (14.81)	9 (24.32)	0.69
Rebleeding day			0.82
1–3	8 (29.63)	10 (27.05)	
4–7	8 (29.63)	9 (24.32)	
8–11	6 (22.22)	11 (29.72)	
>12	5 (18.50)	7 (18.91)	
Multiplex rebleeding			0.44
	3 (11.12)	8 (21.62)	
WFNS scale			0.74
I-II	23 (85.19)	28 (75.68)	
III-IV	4 (14.81)	9 (24.32)	
Fisher scale			0.15
I-II	12 (44.44)	9 (24.32)	
III-IV	15 (55.56)	28 (75.68)	

Syst. BP: systolic arterial blood pressure, Mean BP: mean arterial blood pressure, AComA: anterior communicant artery, MCA: middle cerebral artery, PComA: posterior communicant artery, Carotid. A: carotid artery, IH: intracranial hypertension, Hydroelectrolitic D.: hydroelectrolitic disorders, Sympt. vasospasm: symptomatic vasospasm, and WFNS: World Federation Neurologic Surgeons. ^*^ *P* ≤ 0.05 Chi squares test. % Percent.

**Table 2:** Age, systolic blood pressure, diastolic blood pressure, and serum glucose of rebleeding patients after aneurysmal SAH.

Variables	All patients *n* = 64 Mean (SD)	Survivors *n* = 27 Mean (SD)	Mortality *n* = 37 Mean (SD)	*P* value
Age	50 ± 11.8	52.55 ± 11.87	53.27 ± 13.4	0.597
SBP	144 ± 26.2	141 ± 20.4	151.61 ± 37.2	0.034^*^
DBP	88 ± 13.5	81.55 ± 12.3	88.66 ± 14.5	0.509
Serum Glucose	5.85 ± 1.5	5.61 ± 0.9	7.04 ± 3.3	0.014^*^

^*^ *P* ≤ 0.05 Student *t*-test. SD: standard deviation. SBP: systolic arterial blood pressure; DBP: diastolic arterial blood pressure.

**Table 3:** Risk factors related to mortality revealed by the binary logistic regression analysis.

Mortality	Variable	Categories	*P* value	Odds ratios	95% CI
	Race	Black/Mixed race. White	0.00	4.62	1.40–16.26
Yes/no	SBP	>160 mmHg ≤160 mmHg	0.05	2.54	1.01–3.13
	Serum Glucose	>7.0 mmol/L ≤7.0 mmol/L	0.05	1.82	1.27–2.67

CI: confidence interval. SBP: systolic arterial blood pressure.
